# The validation of online webcam-based eye-tracking: The replication of the cascade effect, the novelty preference, and the visual world paradigm

**DOI:** 10.3758/s13428-023-02221-2

**Published:** 2023-08-30

**Authors:** Ine Van der Cruyssen, Gershon Ben-Shakhar, Yoni Pertzov, Nitzan Guy, Quinn Cabooter, Lukas J. Gunschera, Bruno Verschuere

**Affiliations:** 1 Department of Clinical Psychology, University of Amsterdam, Nieuwe Achtergracht 129B, 1018 VZ Amsterdam, The Netherlands; 2 Hebrew University of Jerusalem, Mount Scopus, 91905 Jerusalem, Israel; 3 Ghent University, Henri Dunantlaan 2, 9000 Ghent, Belgium

**Keywords:** Eye-tracking, Online studies, Cascade effect, Novelty preference, Visual world paradigm

## Abstract

The many benefits of online research and the recent emergence of open-source eye-tracking libraries have sparked an interest in transferring time-consuming and expensive eye-tracking studies from the lab to the web. In the current study, we validate online webcam-based eye-tracking by conceptually replicating three robust eye-tracking studies (the cascade effect, *n* = 134, the novelty preference, *n* = 45, and the visual world paradigm, *n* = 32) online using the participant’s webcam as eye-tracker with the WebGazer.js library. We successfully replicated all three effects, although the effect sizes of all three studies shrank by 20–27%. The visual world paradigm was conducted both online and in the lab, using the same participants and a standard laboratory eye-tracker. The results showed that replication per se could not fully account for the effect size shrinkage, but that the shrinkage was also due to the use of online webcam-based eye-tracking, which is noisier. In conclusion, we argue that eye-tracking studies with relatively large effects that do not require extremely high precision (e.g., studies with four or fewer large regions of interest) can be done online using the participant’s webcam. We also make recommendations for how the quality of online webcam-based eye-tracking could be improved.

Humans are the only species on earth with visible sclera (i.e., the white of the eye; Kobayashi & Kohshima, 1997). Even great apes, which are extremely close to humans in evolution, do not have visible sclera. It has been claimed that the white surrounding human’s darker-colored iris evolved to make it easier for humans to follow the gaze direction of their conspecifics (Kobayashi & Kohshima, 2001). Following others’ gaze is a valuable feature because gaze direction is an indicator of human visual attention (Just & Carpenter, 2018), and events that capture attention for one person could also be relevant for its conspecifics.

What a person is looking at is not only interesting for nonverbal communication in social interactions, but is also useful for exploring more general questions concerning human attention. Since the late twentieth century, video-based eye-trackers have been used to track the eyes in real time (Singh & Singh, 2012) by measuring the position of an infrared light reflection on the cornea (i.e., the transparent layer forming the front of the eye), relative to the pupil (Carter & Luke, 2020). This method allows researchers to track gaze behavior and identify what guides visual attention. In the last 20 years, eye-tracking research gained much popularity and became a common measurement tool in many areas of science (Carter & Luke, 2020).

However, even though eye-tracking research has led to very interesting insights in recent years, this research method has some important limitations. The need for a lab, an expensive eye-tracker device, an experienced researcher who is familiar with the method, and a required calibration procedure make eye-tracking research a rather elaborate, expensive, and time-consuming method. Furthermore, these limitations do not allow for field research in natural environments. These restrictions recently sparked an interest in the use of common webcams to infer the eye-gaze locations of participants (e.g., Bott et al., 2017; Semmelmann & Weigelt, 2018). Moreover, the recent social and economic pressures of the COVID-19 pandemic reinforced this existing interest in webcam-based eye-tracking, as it would allow studies to move online.

The use of a webcam as eye-tracker would make the research quicker, easier, and cheaper, as no lab, experimenter, or dedicated hardware is needed. Moving from lab to web could also allow for reaching a larger and more diverse participant pool more quickly, or reaching a hard-to-reach sample (e.g., patients with dementia; or a US-based researcher wishing to compare US and Chinese participants). The collection of data would no longer be limited by time or location, as individuals could participate whenever they wanted from the comfort of their homes. Importantly, in other fields, research has already shown that the benefits of online research do not necessarily come at a price. Data quality has been shown to be similar to that of lab research (Kees et al., 2017; Walter et al., 2019), and several effects from other fields have already been replicated in online settings (e.g., Dodou & de Winter, 2014; Gosling et al., 2004; Klein et al., 2014; Semmelmann & Weigelt, 2018).

The movement toward online webcam-based eye-tracking research has been facilitated by recent advances in eye-tracking scripts. Open-source eye-tracking libraries such as WebGazer and TurkerGaze enable researchers to use participants’ webcams to infer their gaze position in real time (Papoutsaki et al., 2016; Xu et al., 2015). To do this, the eye-tracking modules build a mapping between the characteristics of the eye (e.g., pupil position) and gaze positions on the screen. The libraries can be easily integrated into any script or experiment with only a few lines of code.

Some studies have already successfully implemented eye-tracking libraries in their online experiments (Semmelmann & Weigelt, 2018; Slim & Hartsuiker, 2021; Yang & Krajbich, 2021). Semmelmann and Weigelt (2018), for example, demonstrated some basic gaze properties (i.e., a fixation task, a pursuit task, and a free viewing task) with online webcam-based eye-tracking. Slim and Hartsuiker (2021), and Yang and Krajbich (2021) both successfully replicated a behavioral eye-tracking experiment (a visual world experiment and a food choice task respectively), although in both studies most participants did not pass the initial calibration/validation phase, and were excluded from the study (73% and 61% exclusions). Moreover, the latter two studies did not directly compare online webcam-based eye-tracking to lab-based eye-tracking. In conclusion, it remains to be established to what extent online webcam-based eye-tracking could be a valid replacement for lab-based eye-tracking and what the cost in terms of capturing cognitive effects on gaze behavior would be.

To validate online webcam-based eye-tracking, we conceptually replicated three classic, robust eye-tracking studies online using the participant’s webcam as an eye-tracker. Furthermore, in the third study, we directly compared the data for participants undergoing both an online webcam-based and a classic lab-based eye-tracking study. Based on Semmelmann and Weigelt (2018), Slim and Hartsuiker (2021), and Yang and Krajbich (2021), we expect to be able to replicate these effects in a web-based setting. Even though some loss of accuracy can be expected, online eye-tracking studies create unprecedented opportunities, as it makes research easier, quicker, and cheaper. This would create great possibilities for studies that require large or hard-to-reach samples or have limited funding. It would also enable the progress of research during pandemic lockdowns.

## Study 1: Cascade effect

The first effect we aimed to replicate was the cascade effect, originally shown by Shimojo et al. (2003). The cascade effect refers to the phenomenon that when people choose which of two presented faces they find most attractive, their gaze is initially distributed evenly between the faces, but then they gradually prioritize the face that they eventually choose. Here, we define the cascade effect as the likelihood of looking at the face that people eventually select during the 100 ms before reporting the decision.

### Method

This study was preregistered (https://osf.io/ykd25). All materials, data, and analytic scripts have been made publicly available and can be accessed at https://osf.io/p3xac/.

#### Participants

An a priori power analysis revealed that for a one-sided one-sample binomial test with an alpha of 0.05, the minimum required sample size was 119 participants to reach 90% power to detect a small effect (*g* = 0.13; which was the observed size of the cascade effect in our pilot study of *N* = 20 when conducting a one-sided one-sample binomial test). Anticipating exclusions, 152 participants were recruited via the online crowdsourcing platform Prolific (https://www.prolific.co). Afterward, we decided that a one-sided one-sample *t*-test would be a more appropriate test. To achieve 90% power to detect a medium effect of *d* = 0.61 (i.e., the effect size of the pilot when conducting a one-sided one-sample *t*-test), we only needed to test 25 participants, which we greatly exceeded in our study. Eligibility was restricted to English-speaking participants with a computer connected to a functioning webcam who did not wear glasses at the time of the experiment and did not participate in the pilot study.

Based on our preregistered exclusion criteria, we excluded five participants for showing no variation in estimated eye gaze across all trials, four for showing no variation in the selected responses across all trials, and seven for having more than 50% of the measurement points falling outside any of the AOIs. Our final sample contained 136 participants (89% of the original sample; 69% male, 31% female, and 0% other) with a mean age of 25 years (SD = 7 years, range 18–48 years). They originated from 28 different countries, of which Portugal had the largest share (24%).

#### Procedure

All participants gave informed consent before taking part in the study. The task was computerized and completed online. In the first part of the experiment, participants provided some demographic information and we double-checked whether they had a working webcam and whether it was placed correctly on their computer. Next, participants saw an instruction screen detailing optimal conditions for webcam-based eye-tracking (see Semmelmann & Weigelt, 2018). Once participants indicated that they had successfully set up according to the instructions, they proceeded to an eye-tracking calibration phase (i.e., participants were instructed that they would see a series of white squares and that they had to look at them and click on them), followed by the main task.

**Fig. 1:**
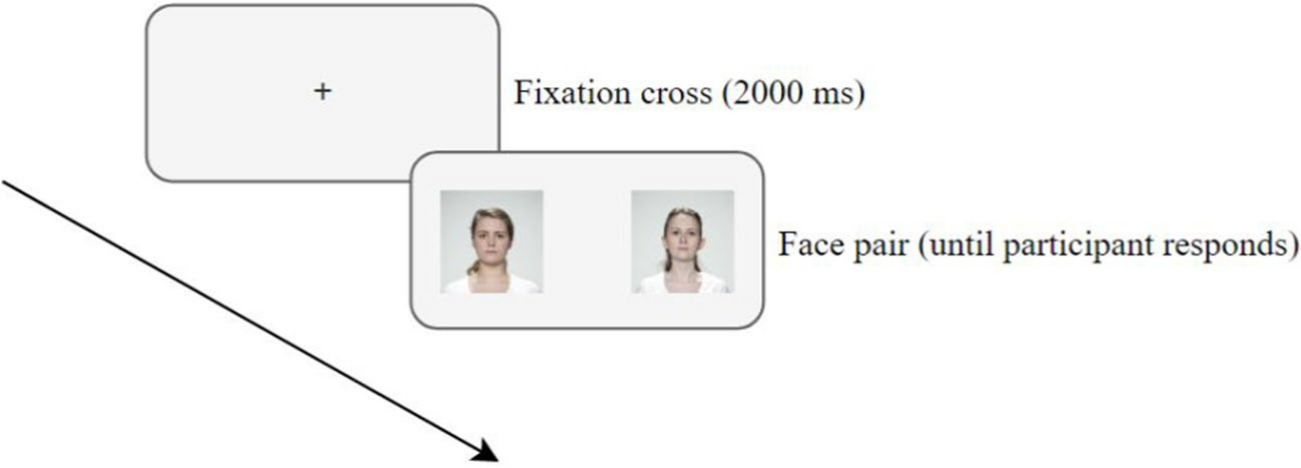
Schematic overview of the course of a trial of the face attractiveness task. Participants were required to select the more attractive face

Each trial of the main task started with a fixation cross (2000 ms), followed by a pair of faces (see Fig. 1). They were instructed to select the one they deemed most attractive by pressing the corresponding key on their keyboard (“F” for the left face, “J” for the right face). They could take as long as they needed to make a decision. There were 18 trials in total. The faces were selected from the London Face Research database (DeBruine & Jones, 2017), which contains images of 102 adult faces with accompanying attractiveness ratings from 2513 individuals. Face pairs were combined based on minimal differences in average attractiveness ratings to replicate the face attractiveness difficult condition of Shimojo et al. (2003). The selected face pairs were matched for gender and ethnicity and were limited to a maximum age difference of 4 years. The order and composition of the face pairs were fixed, meaning that the presentation and the location of each face were consistent across all participants. Faces were presented on a light gray background and vertically centered. The images were 173 × 173 px in size and spaced 295 px apart.

After completing 18 trials, participants received a short debriefing and were thanked for their participation. The demographics and webcam check were programmed in and hosted on Qualtrics (https://www.qualtrics.com), and the eye-tracking part was programmed in PsychoJS and hosted on Pavlovia (https://pavlovia.org/). For the eye-tracking part, we made use of the WebGazer open-source eye-tracking library (Papoutsaki et al., 2016). The entire study was conducted in English.

### Results

#### Preregistered analyses

**Fig. 2:**
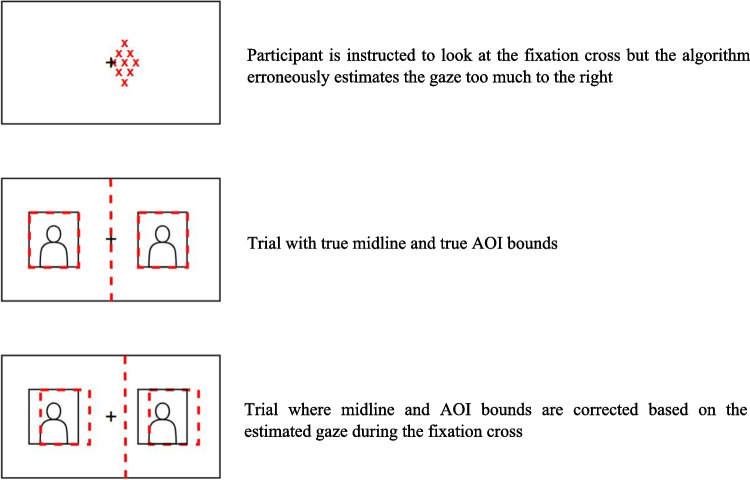
Illustration of how the midline and AOI bounds were corrected based on the estimated gaze during the fixation cross

As previous studies that used online webcam-based eye-tracking lost many participants because they did not pass the initial validation phase, we used an alternative approach in which we manipulated the data after they had been collected, rather than excluding participants who had a too large offset. We extracted gaze position during the tailing 80% of each central fixation period at the beginning of each trial and estimated the measurement bias for that given trial. To account for the offset, the estimated bias was then added to the midline and area of interest (AOI) bounds of that trial (see Fig. 2). For instance, if we found an offset of 50 px to the right of the fixation cross, we would shift the midline and bounds of the AOIs 50 px to the right. As this experiment was limited to a left–right distinction, we applied this correction only for x-values. On average, the midline was corrected for 119 px to either direction (median = 97 px, min = 0 px, max = 480 px). Our confirmatory analyses are based on the midline corrected data. For a comparison between the raw data and the midline corrected data, see our non-preregistered analyses.

Based on our preregistered exclusion criteria, no trials were excluded due to a deviation of the corrected midline of more than 25% of the total screen width from the true midline; no trials were excluded because the standard deviation of the corrected midline was larger than 25% of the screen width; 12% of the measurement points were excluded because they fell outside any of the specified AOIs, and 16% of the trials because the response time was below 0.5 s or above 30 s.

**Fig. 3:**
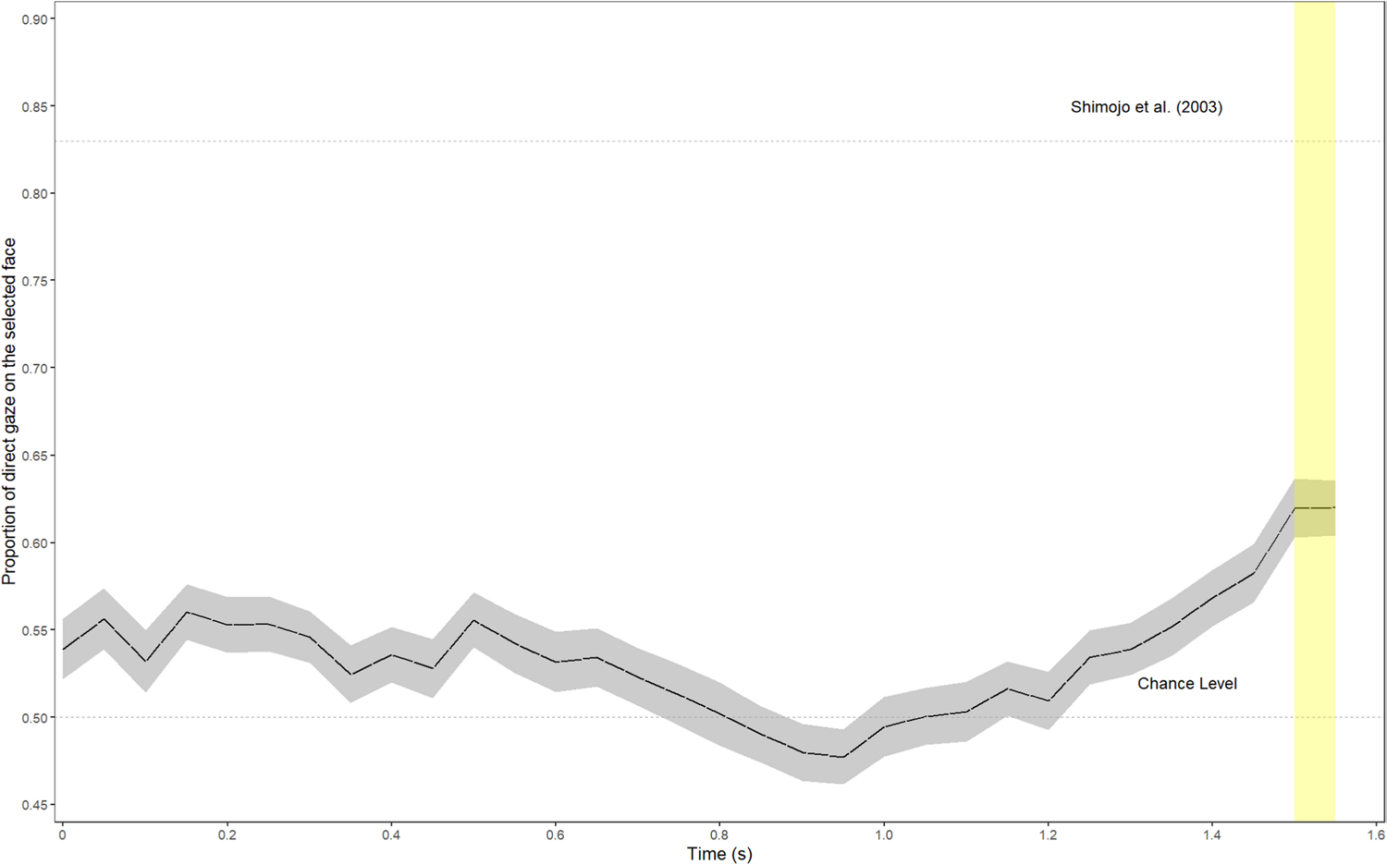
The proportion of time gaze was directed toward the chosen stimulus with respect to the decision time. The period on which we based our analyses is in yellow

Contrary to what we described in the preregistration, we decided that a *t*-test was ultimately a more appropriate test for this study than a binomial test. We did also run all analyses as described in the preregistration, from which the same conclusions were drawn. These analyses can be found at https://osf.io/kwnxc. The results revealed that the likelihood of looking at the face that was eventually selected as more attractive by the participant during the 100 ms leading up to the decision was 62% (as compared to the 50% chance level). A one-sided one-sample *t*-test revealed that this rate was significantly larger than chance, *t*(135) = 7.58, *p* < .001, *d* = 0.65, 95% CI [0.62; ∞]. Additionally, a Bayesian one-sample *t*-test with the default Cauchy scale of 0.707 showed that the data were 3.10 × 10^9^ times more likely under the alternative model in which participants looked more at the eventually chosen face than under the null model of no difference in viewing proportion. This cascade effect is also shown in Fig. 3, which demonstrates a steady increase in viewing proportion over time, and resembles the overall trend reported by Shimojo et al. (2003), although smaller in magnitude (the original study reported an 83% likelihood of looking at the selected face in the 100 ms leading up to the decision).

#### Non-preregistered analyses

##### Midline correction

In the raw data, 7% of the measurement points fell outside of any of the AOIs. In the midline corrected data, 5% of the measurement points fell outside of any of the AOIs. This indicates that we captured slightly more measurement points when adjusting for the offset. When we reran the analyses without the midline correction, we found a 61% likelihood of looking at the face that was eventually selected (compared to 62% with midline correction). This proportion was also significantly higher than chance level, *t*(134) = 7.84, *p* < .001, *d* = 0.67, 95% CI [0.65; ∞]. A Bayesian one-sided one-sample *t*-test showed that the data were 1.19 × 10^10^ times more likely under the alternative model than under the null model.

## Study 2: Novelty preference

The second effect we replicated was the novelty preference effect. This effect refers to the finding that people are more likely to attend to new stimuli than to stimuli they have already seen. This effect is typically demonstrated with the visual paired-comparison task and has been shown by Crutcher et al. (2009), among others.

### Method

The preregistration, materials, data, and analytic scripts of this study are available at https://osf.io/eqg2n/.

#### Participants

An a priori power analysis revealed that for a one-sided one-sample *t*-test (with *α* = 0.05), a minimum sample size of only six participants was required to reach 90% power to detect a large effect (*d* = 1.47; which was observed in our pilot study). To make sure we do have a sufficiently large sample size (as the pilot sample size may not suffice to reliably estimate the true effect size; Brysbaert, 2019), and to account for exclusions, we decided to use a Bayesian stopping rule (Schönbrodt et al., 2017). As described in the preregistration, we first opened the experiment to 50 participants on Prolific. Eligibility was restricted to English-speaking participants with a computer connected to a functioning webcam who did not wear glasses at the time of the experiment and did not participate in the pilot study. Then, after applying the preregistered exclusion criteria (see below), we ran a Bayesian one-sided one-sample *t*-test. The decision to stop collecting data was based on the observed Bayesian factor (BF). We planned that once we reached substantial evidence for either the alternative hypothesis (i.e., BF_10_ larger than 5; participants look more at novel stimuli than what could be expected by chance) or the null hypothesis (i.e., BF_10_ smaller than 1/5; participants do not look more at the novel stimuli), we would stop testing; otherwise, we would open up the experiment for another 50 participants. After our first batch of 50 participants (which ended up being only 49 participants because one participant did not consent to the use of their data), we reached a BF_10_ of 18.16, so data collection was stopped.

Based on the preregistered exclusion criteria, one participant was excluded for showing no variation in estimated gaze position across all trials, and three participants were excluded for having more than 50% of the measurement points falling outside any of the AOIs. The final sample consisted of 45 participants (92% of the original sample; 62% female, 38% male, and 0% other) with a mean age of 27 years (SD = 6 years, range 19–41 years). Participants originated from 14 countries, with the majority having a South African nationality (45%).

#### Procedure

The first part of the procedure was the same as in study 1 (i.e., informed consent, demographics and camera check, instructions about optimal conditions for webcam-based eye-tracking followed by a calibration phase). After this first part, participants proceeded to the main novelty preference task.

**Fig. 4:**
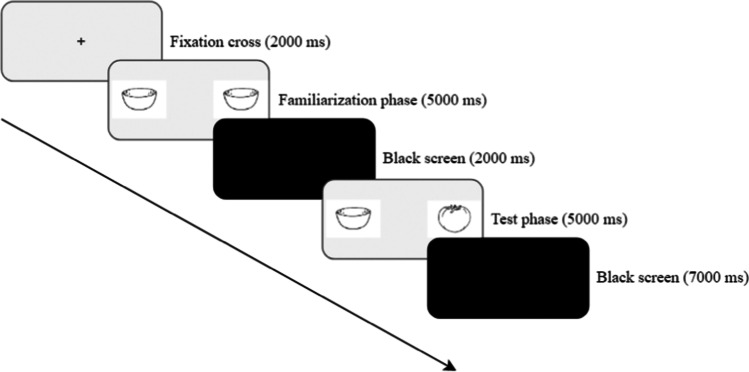
Schematic overview of the course of a trial of the novelty preference task

Each trial of the main task started with a fixation cross (2000 ms), followed by a familiarization phase which consisted of two identical images on the left and right side of the screen (5000 ms). After the familiarization phase, participants saw a black screen (2000 ms), followed by the test phase (5000 ms), in which participants saw two images: one that was the same as the one presented during the familiarization phase and another one that was novel. The left or right positioning of the novel stimulus was randomized across trials. Each experimental trial ended with a black screen (7000 ms; Fig. 4). There were 10 trials in total. Stimuli were black and white, horizontally oriented clipart images selected from the Snodgrass and Vanderwart (1980) database. They were presented on a light gray background, centered vertically, were 472 × 331 px in size, and were 295 px apart from each other.

After the main task, an attention check was performed in which participants were asked to select which of three image pairs they recognized from the experiment. Afterward, they were asked to estimate the reliability of their own data on a five-point Likert scale (with 1 as “unreliable, do not use my data,” and 5 as “very reliable, use my data”). The demographics and webcam check were programmed in and hosted on Qualtrics (https://www.qualtrics.com); the eye-tracking part was programmed in PsychoJS and hosted on Pavlovia (https://pavlovia.org/). For the eye-tracking part, we made use of the open-source eye-tracking library WebGazer (Papoutsaki et al., 2016). The entire study was conducted in English.

### Results

#### Preregistered analyses

In the current study, we corrected the midline on average for 128 px to either direction (median = 122 px, min = 36 px, max = 256 px). Our confirmatory analyses are based on this midline corrected data. For a comparison between the raw data and the midline corrected data, see our non-preregistered analyses. Based on our preregistered exclusion criteria, we excluded 14% of the measurement points because they fell outside any of the specified AOIs; no trials were excluded because the corrected midline deviated more than 25% of the total screen width from the true midline, and 8% of the trials were excluded because the standard deviation of the corrected midline was larger than 25% of the screen width.

**Fig. 5:**
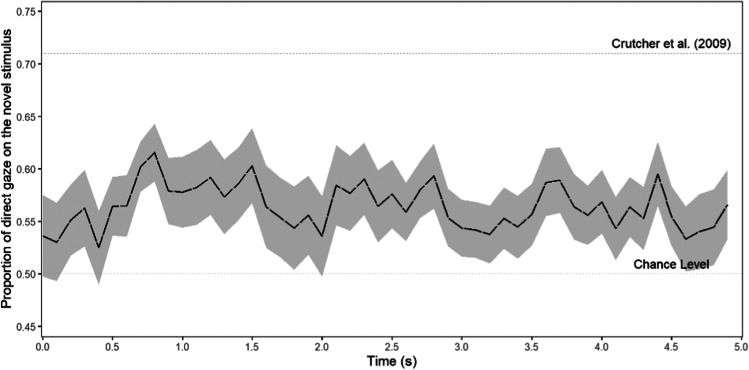
The proportion of time participants looked at the novel stimulus during the test phase of the novelty preference task

On average, participants looked at the novel stimulus 57% of the time (compared to the 50% chance level). A one-sided one-sample *t*-test revealed that this viewing proportion toward the novel stimulus was significantly higher than chance level (.5), *t*(44) = 3.06, *p* = .002, *d* = 0.46, 95% CI [0.42; ∞]. Additionally, a Bayesian one-sided one-sample *t*-test with the default Cauchy scale of 0.707 showed that the data were 18.16 times more likely under the alternative model in which participants looked at the novel stimulus more than 50% of the time, than they were under the null model of no difference in viewing proportion. The effect is visualized in Fig. 5. The results are similar to those reported in previous studies about the novelty preference such as the paper by Crutcher et al. (2009), but with a smaller effect size. For example, in the original study of Crutcher et al. (2009), participants looked at the novel stimulus 71% of the time.

#### Non-preregistered analyses

##### Midline correction

In the raw data, 15% of the measurement points fell outside of any of the AOIs. In the midline corrected data, 14% of the measurement points fell outside of any of the AOIs. When we reran the analyses without performing the midline correction, we found a likelihood of 54% for looking at the novel stimulus (compared to 57% with midline correction). This proportion was still significantly higher than chance level, *t*(44) = 2.05, *p* = .023, *d* = 0.31, 95% CI [0.27; ∞]. A Bayesian one-sided one-sample *t*-test showed that the data were 2.12 times more likely under the alternative model than under the null model.

##### Sensitivity analysis: Can we improve the quality of the data?

In this non-preregistered analysis, we examined whether alternative ways of analyzing the data could improve the results. This could indicate which choices are most consequential for the outcomes. To that end, we sequentially added an increasing number of exclusion criteria so that our data became increasingly strict. First, we excluded participants who failed more than one attention check at the end of the study. Second, we excluded participants who clicked through the instruction screen too fast (i.e., < 7.5 s). Third, we excluded participants who indicated that their data were unreliable at the end of the experiment. Lastly, we excluded measurement points in which the live webcam feed (indicating that the eye-tracker lost the participant’s eyes) appeared. The results can be found in Table 1. Note that these exclusion criteria led to no or very few exclusions, making the results in the different rows very similar.

**Table 1:** Non-preregistered sensitivity analysis

	*N*	Proportion direct gaze	*p*	Cohen’s *d* [95% CI]	BF
Confirmatory analyses	45	57%	.002^*^	0.46 [0.42; ∞]	18.16
+ Attention checks	45	57%	.002^*^	0.46 [0.42; ∞]	18.16
+ Instruction screen	44	57%	.001^*^	0.50 [0.46; ∞]	33.85
+ Unreliable data	44	57%	.001^*^	0.50 [0.46; ∞]	33.85
+ Live feed	44	57%	.001^*^	0.48 [0.44; ∞]	24.77

The table displays the statistical results from the one-sided one-sample (Bayesian) *t*-test for the different exclusion criteria. The exclusion criteria are presented in hierarchical order and include the previous criteria. ^*^ Bonferroni-corrected, statistically significant with *p* < .01

## Study 3: Visual world paradigm

The first two studies of this validation project indicate that it is possible to replicate robust eye-tracking effects with the participant’s webcam while retaining 89–92% of the original sample. However, in both studies, the effect was noticeably smaller than in the original studies. This could be because webcam-based eye-tracking data are noisier, but also because these studies are replications, as it has been demonstrated that the effect sizes of replications are on average 50% smaller than the original effect sizes (Camerer et al., 2018). To estimate how much of the effect size reduction could be attributed to webcam-based eye-tracking, we set up a third study in which each participant conducted the visual world paradigm both online and in the lab with a standard state-of-the-art laboratory eye-tracker (Eyelink 1000 Plus; SR Research Ltd., Mississauga, Ontario, Canada).

We replicated the visual world paradigm effect demonstrating that when people hear utterances while looking at a visual display showing common objects, some of which are mentioned in the sentences, they tend to look more at the images of the words that they hear in the utterances. This effect has been shown by Huettig and Altmann (2005), among others.

### Method

The preregistration, materials, data, and analytic scripts of this study are available at https://osf.io/jucge/.

#### Participants

As described in our preregistration, we first opened the experiment to 50 participants on the participant recruitment site of the University of Amsterdam. Eligibility was restricted to English-speaking participants with a laptop or computer with a functioning webcam and audio device who were not wearing glasses at the time of the experiment. Then, after applying our preregistered exclusion criteria (see below), we ran two Bayesian *t*-tests using the JASP default Cauchy scale of 0.707. First, we ran a Bayesian one-sided one-sample *t*-test on the data of the online version comparing the mean proportion viewing time at the target word to chance level (25%). Second, we compared the mean proportion of viewing time towards the target between the two conditions (lab vs. online), using a Bayesian one-sided paired-sample *t*-test. The decision to stop collecting data was based on the BFs of both *t*-tests. We planned that once we reached substantial evidence for either the alternative hypothesis (i.e., BF_10_ larger than 5) or the null hypothesis (i.e., BF_10_ smaller than 1/5) for both tests, we would stop testing; if not, we would test another 25 participants. We intended to repeat this procedure until we reached substantial evidence in both *t*-tests for either the null or the alternative model or until we had tested *N* = 150. We reached substantial evidence favoring the alternative hypotheses for both models after testing 50 participants.

Based on the preregistered exclusion criteria, no participants were excluded for showing no variation in estimated gaze position across all trials, no participants were excluded for having more than 50% of the measurement points falling outside any of the AOIs, and 18 participants were excluded for having more than 50% missing data (often due to only finishing either the online version or the lab version of the experiment). The final sample consisted of 32 participants (64% of the original sample) that performed both tasks. The sample consisted of 72% females, 28% males, and 0% others and had a mean age of 20 years (SD = 2 years, range 18–27 years). Most participants indicated to be of German nationality (32%).

#### Procedure

This study had a within-subjects design, in which participants completed the experiment both online and in the lab. The order (first online or first in the lab) was counterbalanced. The online part started with a similar procedure as in studies 1 and 2 (i.e., they started with a webcam and audio check, received instructions about optimal conditions for webcam-based eye-tracking, and finished with a calibration phase). In the lab part, we immediately started with the calibration phase as eye-tracking conditions are already close to optimal in the lab. The task in the lab was displayed on a 23-inch Samsung SyncMaster monitor, with a 120 Hz refresh rate and 1024 × 768 screen resolution. Monocular gaze position was tracked at 1000 Hz with an Eyelink 1000 Plus (SR Research Ltd., Mississauga, Ontario, Canada). The participant’s head was stabilized using a chinrest, situated 60 cm from the screen. The experiment started with the standard nine-point calibration and validation procedure provided with the eye tracker. The experiment began if the validation procedure yielded average errors measuring less than 1 degree of visual angle. After the calibration phase, the main task (the visual world paradigm) started, which was identical for the online and lab versions but used different stimuli. The set of stimuli that were used online or in the lab was counterbalanced across participants.

**Fig. 6:**
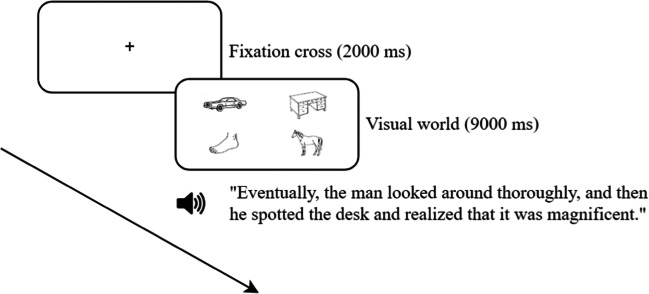
Schematic overview of the course of a trial of the visual world task

Each trial of the main task started with a fixation cross (2000 ms), followed by a screen with four images (e.g., a desk, a car, a foot, and a horse), one in each quadrant of the screen (9000 ms). Each screen with images was paired with a sentence such as “Eventually, the man looked around thoroughly, and then he spotted the desk and realized that it was magnificent.” One of the four images of each scene was a target object that was mentioned in the sentence (in the above example this was the desk), and the other three images (car, foot, and horse) were unrelated distractors (see Fig. 6). Participants were instructed to listen to the sentences carefully and were told that they could look wherever they wanted (they were not asked to perform any task). There was a 1000 ms preview of the display before the onset of the sentence, and the trial was automatically terminated after 9000 ms, which is typically 2000 ms after the end of each sentence. The target word typically appeared 4000 ms after the onset of the sentence. These materials (sentences and scenes) were recreated based on the materials of the experiment of Huettig and Altmann (2005). The images were presented on a white background, were centered on each of the four quadrants of the screen, and had a size of 265 × 189 px. There were 12 trials in each version of the experiment, so 24 trials in total per participant.

After the main task, participants were asked to report how reliable they estimated their own data on a five-point Likert scale (with 1 as “unreliable, do not use my data,” and 5 as “very reliable, use my data”). For the online part, the webcam and audio check were programmed in and hosted on Qualtrics (https://www.qualtrics.com), and the eye-tracking part was programmed in PsychoJS and hosted on Pavlovia (https://pavlovia.org/) in which we made use of the WebGazer open-source eye-tracking library (Papoutsaki et al., 2016). For the lab part, the entire experiment was programmed in Experiment Builder (SR Research Ltd., Mississauga, Ontario, Canada). The study was conducted in English.

### Results

#### Preregistered analyses

In the online part, we corrected the horizontal midline on average for 133 px to either direction (median = 131 px, min = 21 px, max = 265 px), and the vertical midline on average for 134 px to either direction (median = 134 px, min = 48 px, max = 355 px). Our confirmatory analyses are based on the midline corrected data. For a comparison between the raw data and the midline corrected data, see our non-preregistered analyses. Based on our preregistered exclusion criteria, no measurement points were excluded because the live webcam feed indicated that the eye-tracker lost the participant’s eyes, we excluded 15% of the measurement points because they fell outside any of the specified AOIs, we excluded 23% of the trials because the corrected midline deviated more than 25% of the vertical and more than 37.5% of the total screen width from the true midline. Finally, no trials were excluded because the standard deviation of the corrected midline was larger than 25% of the screen width.

 In the lab part, 1% of the measurement points were excluded because they fell outside any of the specified AOIs.

##### Replication of visual world paradigm online with participants’ webcams

The participants looked more at the target items (52%) than the control items (17%) in the online version of the experiment. The proportion of direct fixations on the target items was significantly higher than what could be expected by chance (25%), *t*(31) = 6.39, *p* < .001, *d* = 1.13, 95% CI [1.06; ∞]. Additionally, a Bayesian one-sided one-sample *t*-test showed that the data were 81 572.97 times more likely under the alternative model in which participants looked more at the target item than under the null model of no difference in viewing proportion.

##### Comparison between lab data and online data

**Fig. 7:**
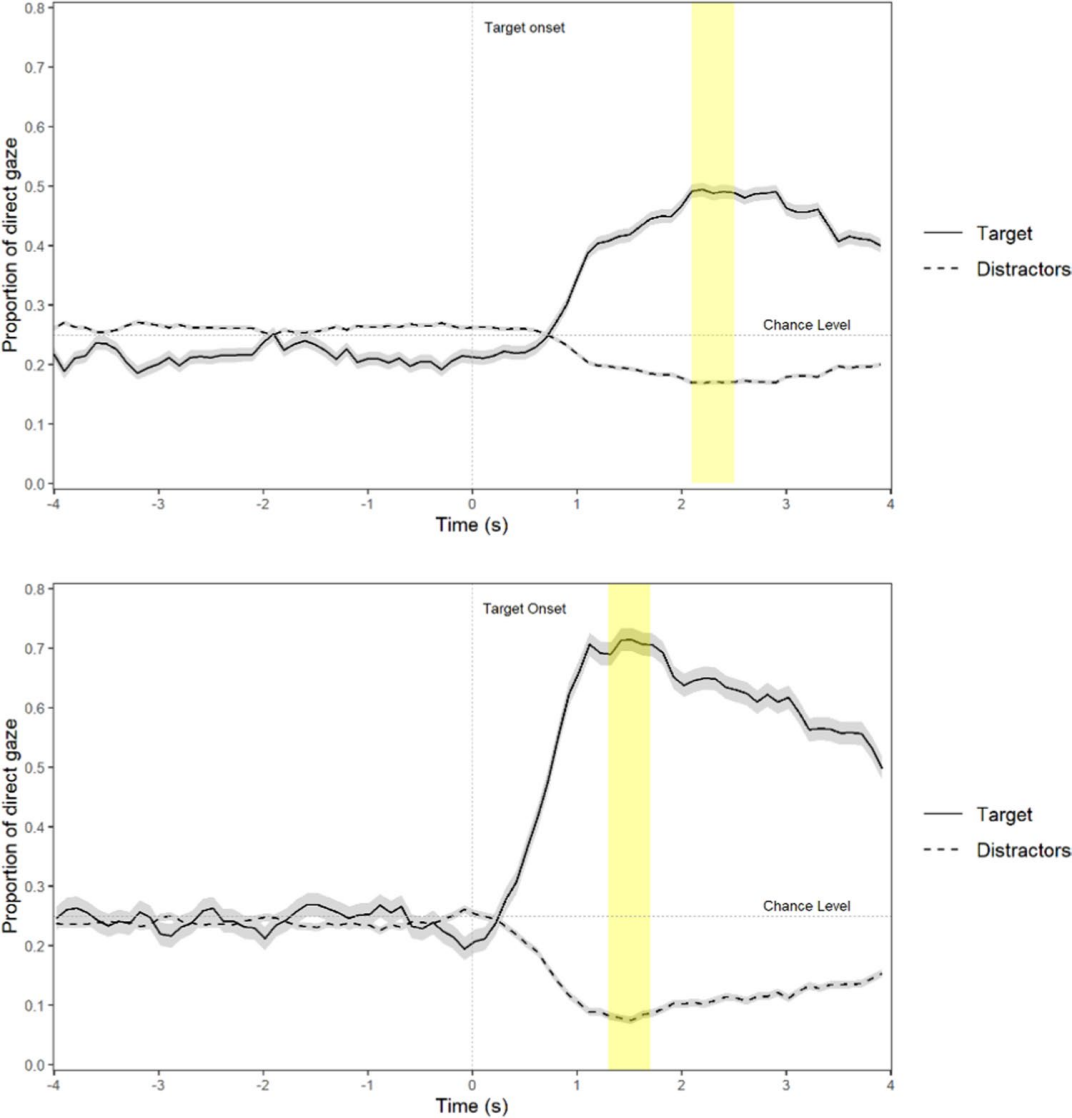
The proportion of time participants looked at the target versus distractors in the online version (top) versus the lab version (bottom) of the experiment. The 400 ms time interval on which we based our analyses is shown in yellow

The proportion of fixations on the target item was higher in the lab version (71%) than in the online version (52%). This difference was significant, *t*(31) = 3.58, *p* < .001, *d* = 0.63, 95% CI [0.54; ∞]. Also, a Bayesian one-sided paired-samples *t*-test showed that the data were 56.75 times more likely under the alternative model (a larger effect size in the lab than in the online version) than under the null model (no difference between lab and web). A visualization of the effect in both the lab version and the web version can be found in Fig. 7.

#### Non-preregistered analyses

##### Midline correction

In the raw data, 15% of the measurement points fell outside of any of the AOIs. In the midline corrected data, 16% of the measurement points fell outside of any of the AOIs. When we reran the analyses without doing the midline correction, we found a likelihood of looking at the target of 38% (compared to 52% with midline correction). This proportion was also significantly higher than the 25% chance level, *t*(32) = 4.11, *p* < .001, *d* = 0.72, 95% CI [0.66; ∞]. A Bayesian one-sided one-sample *t*-test showed that the data were 215.70 times more likely under the alternative model than under the null model.

##### Sensitivity analysis: Can we make the online data more similar to the lab data?

We examined whether alternative ways of analyzing the data could converge the online data to the lab data. This could give an indication of which choices are most consequential for the results. To do this, we added an extra exclusion criterion to the online data making it more strict. We excluded participants who indicated that their data were unreliable at the end of the experiment. The results can be found in Table 2.

**Table 2:** Non-preregistered sensitivity analysis

	*N*	Proportion direct gaze lab	Proportion direct gaze online	*p*	Cohen’s *d* [95% CI]	BF
Confirmatory analyses	32	71%	52%	<.001	0.63 [0.54; ∞]	56.75
+ Unreliable data	31	72%	52%	<.001	0.63 [0.53, ∞]	47.52

The table displays the statistical results from the one-sided one-sample (Bayesian) *t*-test for the confirmatory analyses, and when the exclusion criterium unreliable data is added

##### Calibration score

Inspired by the paper of Slim and Hartsuiker (2021), we calculated the proportion of the estimated gaze position that fell on the center of the screen during a three-second fixation cross immediately after the calibration phase. Similar to Slim and Hartsuiker (2021), we found a mean calibration score of 41% (SD = 26%, range 7–93%) in the online webcam-based data. This was considerably lower than the lab-based data, where the mean calibration score was 99% (SD = 4%, range 75–100%). To test whether higher calibration scores could lead to larger effect sizes in the online webcam-based data, we calculated the Pearson correlation between the calibration scores and effect sizes of the online webcam-based data. However, this analysis showed no relation between the two (*r* = −0.04, *p* = .843).

## General discussion

Can we validly run eye-tracking studies with participants’ own webcams? We replicated three robust eye-tracking studies (the cascade effect, the novelty preference, and the visual world paradigm) online with the participant’s webcam as eye-tracker. All three studies were replicated successfully, and the research was conducted considerably faster, easier, and cheaper than comparable studies conducted in the lab. To illustrate, data collection for studies 1 and 2 was completed within 1 day, and participants from 14 to 28 different countries took part. Moreover, contrary to previous online webcam-based eye-tracking studies, we could retain most of our sample (64–92%). This indicates the potential of an online webcam-based eye-tracking procedure that captures gaze directed to left\right or four quadrants of the screen. However, even though the overall effects were replicated, the effect sizes of all three studies shrank by 20–27% as compared to lab-based eye-tracking.

### Smaller effect sizes

There are several reasons that could explain why the effect sizes of the online webcam-based studies were smaller than those of lab-based eye-tracking studies. For example, it is possible that webcam-based eye-tracking leads to an underestimation of the true effect size. This could be due to smaller numerators (i.e., if the mean differences between the test values of online webcam-based eye-tracking are smaller) or larger denominators (i.e., if the standard deviations of online webcam-based eye-tracking are larger). The third study of the current paper revealed both that the numerator of online webcam-based eye-tracking was 41% smaller than that of lab-based eye-tracking, and that the denominator was 32% larger.

At the same time, original effect sizes often overestimate the true effect sizes. It has been shown that the effect sizes of replications are typically 50% smaller than the effect sizes of original studies (Camerer et al., 2018). It is argued that this is caused by exaggerated effect size estimates in the existing literature due to a combination of publication bias and questionable research practices (e.g., Simmons et al., 2011; Sterling, 1959). In the third study of the current paper, we found the effect size of the lab-based replication indeed to be lower than that of the original study. However, the effect size of the online webcam-based replication was even lower than that of the lab-based replication, so replication per se could not fully account for the effect size shrinkage in online webcam-based eye-tracking. This indicates that the decreased effect sizes of online webcam-based eye-tracking are probably caused by a combination of both factors.

As in other conceptual replications (i.e., replications where there are changes to the original procedures), the decrease in effect size could also be related to several methodological differences between the original studies and our replications (Zwaan et al., 2018). First, while all original studies were conducted in the lab, which is a very controlled environment, all webcam-based replications were done online. Second, as we did not have the original materials for studies 1 and 2, we intuitively reconstructed them ourselves. Third, we used different measurement tools. The original studies used standard laboratory eye-trackers; we used webcams. It is possible that we did not measure the exact same construct as the original studies because of these changes, which could result in different effect sizes.

### Lower data quality

In the context of eye movement experiments, data quality refers to the extent to which the collected eye-tracking data accurately and reliably reflect the participants’ visual behavior (Holmqvist et al., 2012). To ensure data quality in eye-tracking experiments, researchers typically employ various strategies such as calibration and validation procedures, adherence to standardized guidelines for the setup of the eye-tracker and environment, consistent monitoring during data collection, and preprocessing techniques (e.g., noise reduction, outlier removal; Holmqvist et al., 2011). Many of these strategies are not evident for online webcam-based eye-tracking due to the less controlled environment of online testing. Moreover, the quality of webcam hardware is known to be lower than cameras used in standard eye-trackers. Because of these reasons, the data quality of online webcam-based eye-tracking is expected to be lower than that of lab-based eye-tracking. We tested whether this quality could be improved in two ways: midline correction and stricter exclusion criteria.

#### Midline correction

The use of validation trials is a common method of correcting for deterioration of gaze estimation accuracy. Although effective, they can take up large proportions of the experimental duration, and it has been debated how much validation is required to ensure high gaze estimation accuracy (e.g., Semmelmann & Weigelt, 2018). For example, in previous studies that used online eye-tracking, the validation phase took up to 40–50% of the experimental duration (Semmelmann & Weigelt, 2018; Yang & Krajbich, 2021). Moreover, only 27– 39% passed the initial validation phase in the studies of Slim and Hartsuiker (2021) and Yang and Krajbich (2021). In the current study, we tested an alternative approach that involves manipulating the data after they have been collected, rather than monitoring the offset throughout the experiment and excluding participants based on this offset (Hornof & Halverson, 2002). This approach has some important benefits such as cutting back on computational demands and experimental duration, and losing fewer participants during the validation phase. Based on the systematic measurement error estimates from the fixation period preceding each trial, we shifted the midline and AOI bounds to the respective direction. Our non-preregistered analyses showed that the effect sizes of the midline corrected data were higher than those of the raw data, suggesting a successful improvement in data quality.

#### Stricter exclusion criteria

It has been argued that exclusion criteria and attention checks can greatly improve the quality of the data. In our non-preregistered analyses, we tested whether applying stricter exclusion criteria could improve data quality and decrease the differences between online webcam-based eye-tracking and lab-based eye-tracking. However, attention checks did not seem to make large differences in our study. Participants were largely attentive, which led to few or no exclusions based on the criteria. This is in line with the literature indicating online participants to be of similar quality to those acquired via university pools (e.g., Goodman & Paolacci, 2017; Gould et al., 2015). However, our exclusion criteria were merely focused on inattention and the live webcam feed (indicating that the eye-tracker lost the participant’s eyes). Checks for poor environmental circumstances (e.g., too much light, too little light, poor webcam quality, slow internet) might have been more successful in improving data quality.

In sum, in all three studies, we were able to slightly improve the data quality. However, in all three studies, the conclusions remained exactly the same with or without midline correction, and with or without stricter exclusion criteria. Furthermore, higher calibration scores did not seem to predict higher effect sizes. This shows that extensive validation phases, data manipulation, and extreme exclusion criteria might not be necessary to reach valid results. Excluding more participants and measurement points might improve the estimated effect size, but might also lead to higher costs and a longer process. Furthermore, hard-to-reach samples are likely excluded in lab-based experiments. The ideal trade-off between data quality and data exclusion/manipulation probably depends on the specific study characteristics and study goal.

### Practical considerations for online webcam-based eye-tracking

Determining the suitability of an eye-tracking study for online webcam-based data collection depends on several factors. For instance, studies that involve eye-tracking effects that are known to be stable and robust, such as the replication studies presented in the current paper, are strong candidates for web-based eye-tracking. Additionally, new experiments that are expected to have large effects and a limited number of large areas of interest could be effectively conducted using web-based eye-tracking methodologies. This way, webcam-based eye-tracking could be used for both original and replication studies. For example, studies exploring attentional orienting, such as investigations into the spatial cueing effect, research on social attention like face perception studies, or research on memory and visual attention such as change detection studies, could potentially benefit from online webcam-based eye-tracking. These studies often involve a limited number of large areas of interest and global gaze measures, which aligns well with the capabilities of the webcam-based setup.

On the other hand, studies requiring high precision and accuracy, such as those involving small or intricate areas of interest, demanding fine-grained temporal measurements, or investigating subtle eye movements such as blinks, saccade trajectories, fixation durations, or pupil dilatations, may still necessitate traditional laboratory-based eye-tracking systems. For example, studies focusing on psycholinguistics and language processing, such as investigations of sentence reading or word recognition, may be less suitable for webcam-based eye-tracking. It is crucial for researchers to evaluate the specific requirements of their study, considering the precision and level of detail needed, to determine the appropriateness of online webcam-based eye-tracking.

When opting for online webcam-based eye-tracking, researchers should consider several best practices. First, determining an appropriate sample size and conducting a power analysis remain crucial steps in ensuring statistical robustness. However, as the effect sizes of online webcam-based eye-tracking are relatively small with large variability among participants, sample sizes will need to be larger than in traditional studies. We suggest anticipating a 20–30% reduction in the expected effect size in the power calculation. Second, it is important to provide detailed instructions to the participant regarding the ideal experimental setting, and where possible valid ways to check adherence to the instructions. This includes guidelines such as avoiding head movements and indicating a suitable distance from the screen and luminance in the room. These instructions could help compensate for the less controlled environment of online testing. Third, researchers will need to establish clear criteria for participant and data exclusion to maximize data quality. For shorter experiments, calibration and validation procedures can be used, while for longer experiments, midline correction may be a more reasonable alternative. Additionally, conducting pilot and exploratory preliminary studies can help identify effective checks to facilitate the exclusion of uncooperative participants or participants with environments unsuitable for eye-tracking.

To end on a positive note, webcams, cameras, and processing systems are rapidly improving. Over time, the described limitations of online webcam-based eye-tracking will become less significant, the method will become feasible for a greater number of studies, and fewer requirements will have to be met to still obtain high-quality data.

### Limitations of the current study and moving forward

The experiments in the current paper were quite simple; they only took 5–7 min and only had 2–4 AOIs. It would be interesting to investigate how far the effects could be pushed and how many distinct AOIs could be effectively used online. Based on WebGazer’s spatial precision, Yang and Krajbich (2021) suggested that up to six AOIs could be used without any degradation in data quality. Their study took 30 min and with several re-calibrations they were able to maintain appropriate data quality. For longer studies or studies having more than six AOIs, we hypothesize that more re-calibrations might be needed.

Once the groundwork has been established, conceivable applications of online webcam-based eye-tracking are numerous. Among other benefits, the possibility to reach difficult-to-access populations is a major advantage for online versus lab-based research. For instance, eye-tracking has been found valuable for predicting the onset of Alzheimer’s disease (Bourgin et al., 2018; Crawford et al., 2015; Crutcher et al., 2009). Potentially conducting this or similar assessments from the comfort of one’s home not only would save resources and reduce interpersonal contact, but would enable thousands of (at-risk) patients to be reached, broaden the availability of eye-tracking methodology (even during pandemic lockdowns), and change the way we conduct eye-tracking research in general.

## Conclusions

Our study provides evidence for the applicability of online webcam-based eye-tracking. In three replications of robust eye-tracking studies, we demonstrated that similar findings could be obtained when doing the study online with the participant’s webcam as eye-tracker. While the conclusions are limited by the simplicity of our tasks, these replications collaboratively serve to inform gold standards for the application of online webcam-based eye-tracking. Although the data quality was considerably lower, the speed and convenience of online research could be worth the switch for studies with large effect sizes and relatively few AOIs. Moreover, with the ever-improving quality of computer webcams, the future of online eye-tracking looks promising.
